# Hit integration for identifying optimal spaced seeds

**DOI:** 10.1186/1471-2105-11-S1-S37

**Published:** 2010-01-18

**Authors:** Won-Hyoung Chung, Seong-Bae Park

**Affiliations:** 1 Department of Computer Engineering, Kyungpook National University, Daegu 702-701, South Korea

## Abstract

**Background:**

Introduction of spaced speeds opened a way of sensitivity improvement in homology search without loss of search speed. Since then, the efforts of finding optimal seed which maximizes the sensitivity have been continued today. The sensitivity of a seed is generally computed by its *hit probability*. However, the limitation of hit probability is that it computes the sensitivity only at a specific similarity level while homologous regions usually distributed in various similarity levels. As a result, the optimal seed found by hit probability is not actually optimal for various similarity levels. Therefore, a new measure of seed sensitivity is required to recommend seeds that are robust to various similarity levels.

**Results:**

We propose a new probability model of sensitivity *hit integration *which covers a range of similarity levels of homologous regions. A novel algorithm of computing hit integration is proposed which is based on integration of hit probabilities at a range of similarity levels. We also prove that hit integration is computable by expressing the integral part of hit integration as a recursive formula which can be easily solved by dynamic programming. The experimental results for biological data show that hit integration reveals the seeds more optimal than those by PatternHunter.

**Conclusion:**

The presented model is a more general model to estimate sensitivity than hit probability by relaxing similarity level. We propose a novel algorithm which directly computes the sensitivity at a range of similarity levels.

## Background

Finding homologous region is one of the most important tasks in current biological works. It is a task to find similar regions between biological molecular sequences. This task is translated into local alignment which scores the similarity between sequences with an edit distance. The state-of-the-art scoring method for the local alignment is *Smith-Watermann *dynamic programming algorithm [1]. The main drawback of this algorithm is that it is impractical with large-scale search tasks due to its high computational complexity.

One practical approach to speeding up local alignment algorithms is filtering out non-homologous regions before aligning sequences. This process consists of a filtering step and an extension step in general. At the filtering step, short fixed-length common words that are found at both query and target sequences are selected. Then at the extension step, it is determined whether each word can be extended into a significant alignment. BLAST [2] and FASTA [3] are the most popular programs among the filtering-based programs for homology search. Both of them use fixed-length continuous matches as a template for finding common words, and the template is called a *seed*. This approach has a drawback of sacrificing sensitivity for speed. The bigger the seed size is, the higher the risk of missing true alignments gets. At the same time, small-sized seeds tend to generate more random hits, and then result in computational slowdown. PatternHunter [4] enabled more sensitive homology search than BLAST by introducing a non-continuous seed such as 111*1**1*1**11*111, so-called a *spaced *seed.

After the notion of non-consecutive seed was presented, the spaced seed has been studied by many researchers in aspects of computational complexity. The studies of evaluating the performance of spaced seeds over consecutive seeds were investigated by [5-9]. Among these researches, [8] and [9] showed that finding the optimal spaced seeds is NP-hard, but the sensitivity of a seed can be computed in polynomial time. Several algorithms [5-7,10-12] have been proposed to compute the sensitivity of a seed exactly. In addition, these algorithms can be accelerated by several heuristic methods [10,13-19]. The extension of spaced seeds was achieved by adapting the seeds for more specific biological sequences [20-24] or building models to understand the mechanism which makes spaced seeds powerful [14,22,25,26].

Although many advanced evaluation measures are proposed as measures of spaced seed's sensitivity, *Hit probability *proposed by Ma et al. [4] is still being used as a notion equivalent to the seed's sensitivity. The hit probability of a seed is defined as a probability of finding the seed at a random sequence at a specific similarity level. The limitation of hit probability is that it is computed only at a specific similarity level even though homologous regions actually have various similarity levels. As a result, an optimal seed for a specific similarity level could not be optimal for other similarity levels. For instance, the seed presented by PatternHunter (111*1**1*1**11*111) was only optimal at the range of similarity levels from 61% to 73% [10]. However the homologous regions of genome comparison have more diverse range of similarity levels. Therefore, a new measure of seed's sensitivity is needed which covers a range of similarity levels of homologous regions.

This paper proposes an algorithm of computing integration of hit probabilities at a range of similarity levels. In order to reflect a range rather than a point, the integration of hit probabilities is used, and it is named as *hit integration *since it is considered as an accumulation of hit probabilities. We also prove that hit integration is computable by expressing the integral part of hit integration as a recursive formula which can be easily solved by dynamic programming. The experimental results show that hit integration reveals the seeds more optimal than those by PatternHunter. The seed 111**1*11**1*1*111 is found to be an optimal seed for hit integration at the range of similarity levels from 0 to 1. This seed results in about 2% higher sensitivity than PatternHunter seed.

The rest of this paper is organized as follows. Hit integration is first introduced and dynamic programming algorithm to compute hit integration is presented formally. Next, the optimal seeds under hit integration is compared with the seeds recommended by other probability models. Then, the quantitative comparison between seeds is explained. In Methods after conclusion, the way of the experiment using biological data follows.

## Results and discussion

### Hit integration

Hit probability of a seed is defined as the probability of finding the seed at a *L*-length bernoulli random sequence at a similarity level *p*. It has been used as a synonym of the sensitivity of a seed. However, hit probability is tightly linked with a specific value of similarity level, whereas sensitivity refers to the probability that a seed hits a region without consideration of the similarity level *p*. We define a more general probability model to measure sensitivity than hit probability by extending a specific value of similarity level to a range of similarity levels.

#### Definition of hit integration

**Figure 1:**
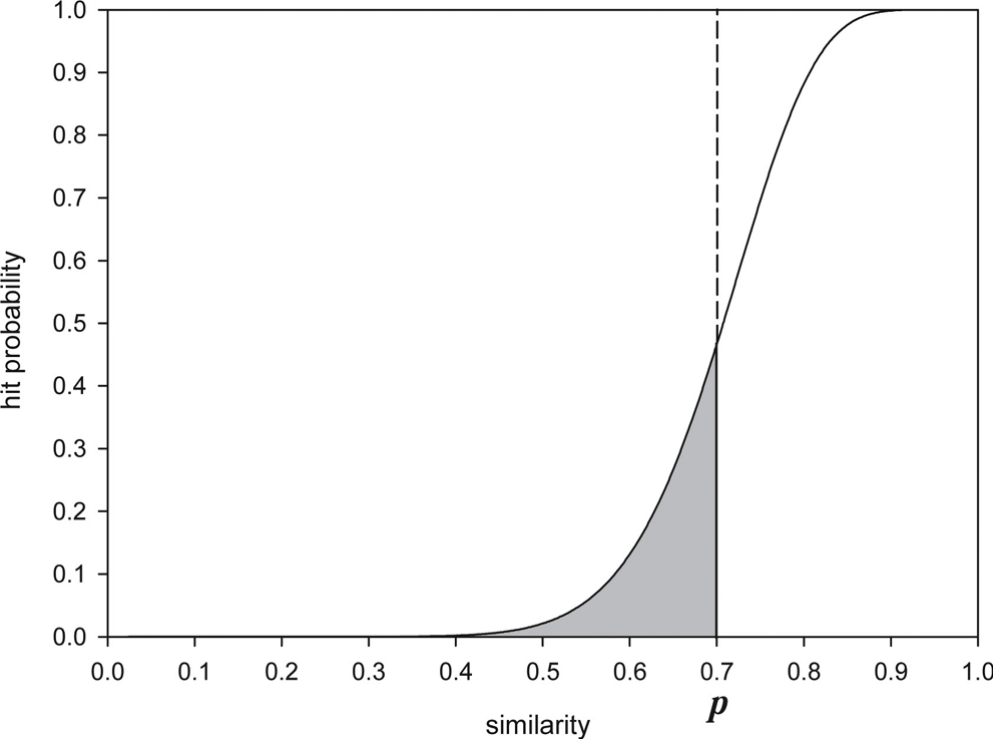
**An illustration of hit integration**. Solid line and dashed line stand for the curve of hit probability and hit integration, respectively. Gray area underneath the curve of hit probability implies the amount of hit integration at *p*. *p *is the upper limit of hit integration.

As the sensitivity covers a range of similarity levels, *hit integration *is defined to be the integration of hit probabilities over a range of similarity levels. The cumulation of the hit probabilities over a range of similarity levels is illustrated in Figure 1. The gray area below the curve represents integration of the hit probabilities from similarity level 0 to *p*. It indicates clearly hit integration of a seed below a similarity level *p*. Therefore, hit integration can be computed by integration of hit probabilities. In order to simplify the expression of hit integration, we denote hit integration over a range of similarity levels from *a *to *b *as *HI *[*a, b*] where 0 ≤ *a *<*b *≤ 1. Because the presented algorithm in this paper can compute the hit integration from similarity level 0 to *p*, hit integration for an arbitrary range of *a *and *b *is defined as below. Given both *HI *[0, *a*] and *HI *[0, *b*], *HI *[*a*, *b*] is computed by (*HI *[0, *b*]*HI *[0, *a*])/(*b *- *a*). A special case of hit integration is when it covers all similarity levels, from 0 to 1. In this case, the similarity parameters *a *and *b *are not needed. We will call it as *similarity-level-free *sensitivity.

### Computing hit integration

The computing algorithm for hit integration is derived from the dynamic programming algorithm of hit probability. At least to our knowledge, it is impossible to obtain an integral form directly from the hit probability because of its recursive form. We propose a novel idea of integrating the recursive form. Before describing our novel algorithm, we briefly describe the algorithm to compute hit probability proposed by Keich et al. [7]. And then extend the algorithm to compute hit integration.

#### Notations

The notations for defining hit integration follow from those in [12]. A spaced seed *Q *of which length is *m *is a string consisting of 1's and *'s. 1 stands for a 'require match' and * for a 'don't care'. The number of 1's in *Q *is called as its *weight *denoted as *w*. Conventionally, the first and last letter of *Q *are defined as 1. For example, the length and the weight of a seed 111*1**1*1**11*111 are 18 and 11 respectively. From this point on, a seed is assumed to be a spaced seed for convenience. A homologous region *R *with a length *L *is represented as a binary string in which 1 stands for a match and 0 for a mismatch. *R *[*i*:*j*] represents a subregion of *R *from *i*-th to *j*-th position. We assume that *R *is a Bernoulli random string. Then, the probability of occurrence of 1 in a position *R *[*i*] is denoted as *p*, and this *p *is called as the similarity level of *R*. For a seed *Q *and a homologous region *R*, *Q *is said to *hit R *if *R *has a substring *R *[*i*:*i *+ *m - *1] which satisfies *R *[*i *+ *j*] = 1 whenever *Q *[*j*] = 1. Given a homologous region *R *with a length *L *and a similarity level *p*, the *hit probability *of a seed *Q *is the probability that *Q *hits *R *at or before the position *L*.

#### Computing hit probability

For a seed *Q*, a string *b *is said to be *compatible with Q *if *b *[|*b*| - *j*] = 1 when *Q *[*m - j*] = 1 for all 0 ≤ *j *≤ min(*m*, |*b*|). Let *B *be a set of all *b*'s compatible with *Q*. For a string *b *∈ *B*, let *f*(*i, b*) be the probability that *Q *hits region *R *[1:*i*] which has *b *as its suffix. In case of |*b*| = *m*, *f*(*i*, *b*) is 1 since *Q *always hits *b *if *b *belongs to *B*. That is,(1)

In the cases where *i *is smaller than *m*, *f*(*i, b*) is always 0 since *Q *can not hit any region shorter than |*Q*|. Thus,(2)

When 0 ≤ |*b*| ≤ *m*, there are two cases: (i) the position before *b *has value 0, and (ii) the position before *b *has value 1. Thus, *f*(*i, b*) can be recursively expressed as(3)

The hit probability of *Q *on *R *is then equal to *f *[*L*, ϵ], where *L *is the length of *R *and is an empty string. That is, *f *[*L*, ϵ] is the probability that *Q *hits the region *R *when a suffix is not given. The suffix 1*b *is always within *B *if |1*b*| ≤ *m*, but the suffix 0*b *could not be within *B*. Keich et al. [7] showed that *f *[*i*, 0*b*] is equivalent to *f *[*i*, 0*b*'], where *b*' is the longest prefix of an element in *B*. When *B*(*x*) is the longest proper prefix of a string that is in *B*, this relation is written as follows.(4)

#### Computing hit integration: the integration of hit probabilities

*HI *[0, *p*] is the integration of hit probabilities of similarity levels from 0 to p where 0 ≤ *p *≤ 1. The integral form is derived from the recursive function of hit probability by following modifications. According to Equation (3), the integration of *f *[*i, b*] can be represented as a linear combination of other integral forms.(5)

Let *I*^*n *^[*i, b*] be an integral of the multiplication of *p*^*n *^and the hit probability function *f *[*i, b*], where *n *is the degree of the function *I*. That is,(6)

*I*^0 ^is equivalent to the integration of hit probabilities. Therefore, *I*^0 ^with length *L *and an empty suffix ϵ gives the exact solution of the integral of hit probabilities. From the equation *I*^0 ^= ∫ *f*[*i*, *b*]*dp *and Equation (5), Equation (6) is expanded as(7)

This function corresponds to Equation (3). Equation (1), Equation (2), and Equation (4) are also translated into the following forms.(8)(9)(10)

Mathematical proofs of Equation (7)~(10) are described at Additional file 1.

### Dynamic programming of hit integration

The dynamic programming algorithm for hit integration is obtained from the dynamic programming of hit probability by substituting Equation (7)~(10) for Equation (1)~(4). The algorithm to compute the hit integration of a seed *Q *is given below. The goal of this algorithm is to find *I*^0 ^[*L*, ϵ], since it is equivalent to *HI *[0, *p*].

**Input **Seed *Q*, Positive probability *p*, Length *L *

**Output **Hit Integration of *Q*

1. Compute the suffix set *B *compatible with *Q*.

2. **For ***i *= 0 **to ***L ***do**

3.   **For ***b *∈ *B *from largest to shortest **do**

4.      *n*_*min *_= the number of 1's in *b*

5.      *n*_*max *_= *L - i *+ |*b*|

6.      **For ***n *= *n*_*min *_**to ***n*_*max *_**do**

7.         **If ***i *< |*b*| **Then**

8.            *I*^*n *^[*i, b*] = 0

9.         **Else If **|*b*| = *m ***Then**

10.            

11.         **Else**

12.            *I*^*n *^[*i*, 0*b*] = *I*^*n *^[*i *- |*b*| + |*b'*| 0*b'*] where 0*b' *= *B*(0*b*)

13.            *I*^(*n*+1) ^[*i*, 0*b*] = *I*^(*n*+1) ^[*i *- |*b*| + |*b'*|, 0*b'*] where 0*b' *= *B*(0*b*)

14.            *I*^*n *^[*i, b*] = *I*^(*n*+1) ^[*i*, 1*b*] + *I*^*n *^[*i*, 0*b*] - *I*^(*n*+1) ^[*i*, 0*b*]

15.         **End If**

16.      **End For**

17.   **End For**

18. **End For**

19. **Return ***I*^0 ^[*L*, ϵ]

The suffix set *B *need to be obtained before entering the first loop at line 2 since *B *is not changed during the execution of the algorithm. *B *can be easily obtained by getting all combinations of binary suffix strings. All strings *b *∈ *B *have to meet the following constraints. For all 0 ≤ *i *≤ |*b*|, *b *[*i*] = 1 if *Q *[*i*] = 1, and *b *[*i*] is 0 or 1 if *Q *[*i*] = 0. For each suffix *b*, its longest prefix *B*(0*b*) need to be computed in advance. The computational procedure for *B *is same as that in the dynamic programming for hit probability. Thus, the details of computing *B *is skipped in this paper. Refer to [7] for this procedure.

The algorithm has three nested loops. Line 2 is the outer-most loop which increases the region length parameter *i *from 0 to *L*. The second loop at line 3 considers all suffixes *b *∈ *B*. This loop also runs in descending order from the longest to the shortest suffix, an empty string. Line 4 and 5 are the bound of all possible degrees of a function *I *where *i *and *b *are given. The minimum bound of the degree is limited to the number of 1's in a suffix *b*. The maximum bound of the degree is limited to the size of *L - i *+ |*b*|. Line 6 is the inner-most loop for a degree of *n *which computes *I*^*n*^. Line 7~8 compute Equation (9), and line 9~10 compute Equation (8). At line 12~13, *I*^*n *^for 0*b *and *I*^(*n*+1) ^for 0*b *are obtained from the previously computed *I*'s by following Equation (10). Line 14 computes *I*^*n *^[*i, b*] using Equation (7). This algorithm finally returns *I*^0 ^[*L*, ϵ] at line 19.

#### Time complexity

The following proof will show that hit integration can be computed exactly with only *L *additional time in compare with hit probability. Time complexity of this algorithm is derived by the loops of three variables, *i*, *b*, and *n*, and the precomputation time of the suffix set *B*, the longest prefix set *B*(0*b*), and all possible values of Equation (8). The number of all elements of *B *is  where *b*_*w *_is the number of 1's in a suffix *b*, and |*b*| is the length of suffix *b*. It is bounded by *m*·2^(*m *- *w*) ^where *m *is the length of the seed *Q *and *w *is the weight of *Q *since the maximum length of *b *is equal to the length of *Q*. Therefore the suffix set *B *at line 1 is computed at *O*(*m*·2^(*m *- *w*)^). All possible values of Equation (8) at line 10 can be computed within *L *+ *m *times because it is bounded by the variable *n *and its maximum value is *L *+ *m*. For all *b*, the longest prefix *B*(0*b*) at line 12 and 13 can be computed in advance in time *O*(*m*^2^·2^(*m *- *w*)^). The time needed to compute the three nested loops at line 2, 3, and 6 is *O*(*L*·*m*·2^(*m *- *w*)^·(*L *+ *m*)). The number of iterations of the outer-most loop for variable *i *at line 2 is *L *+ 1. Since the loop for variable *b *at line 3 iterates the number of all elements of *B*, it runs at most *m*·2^(*m *- *w*)^times. The innermost loop for variable *n *at line 6 iterates *L *+ *m *times. Notice that the time complexities of all precomputations are less than the time complexity of the loops. Therefore, the total time complexity of the algorithm is *O*(*L*^2^·*m*·2^(*m *- *w*)^). Because the time complexity of dynamic programming of hit probability is *O*(*L*·*m*·2^(*m *- *w*)^) [7], hit integration can be computed exactly with only *L *additional time in compare with hit probability.

#### Validity of the proposed algorithm

The proposed dynamic programming can be validated by comparing it with another accumulation methods, Riemann sum and Gaussian quadrature. Riemann sum is a well-known method for approximating the total area underneath a curve on a graph [27]. Gaussian quadrature is also well-known approximation method of the definite integral of a function, usually stated as a weighted sum of function values at specific points within the domain of integration [28].

Thus, for the validity of the proposed algorithm, *HI *[0, *p*] is compared with Riemann sum of hit probabilities. If a range [0, *p*] is partitioned into *N *sub-ranges, Riemann sum of hit probability is defined as

where *p*_*i *_is a similarity level within [0, *p*]. The difference between the outcome of hit integration and Riemann sum is averagely lower than 10^-5 ^when *N *is set to 1000. For instance, the Riemann sum of the hit probability for the default seed of PatternHunter at the range from 0 to 1 is 0.300029 whereas the hit integration is 0.300031. This small difference is believed to be made by the approximation error of the Riemann sum.

The proposed algorithm is also compared with Gaussian quadrature of hit probabilities. When a range [0, *p*] and the sequence length *L *are given, we compute *L*-point Gaussian quadrature where lower limit is 0 and upper limit is *p*. First, the weights and similarities are calculated for *L *points. And then all integrands are computed by multiplying the corresponding weights after computing hit probabilities for the similarities, respectively. The integration is the sum of the integrands. The approximation of integration using Gaussian quadrature gives nearly identical result by comparing with the result of hit integration. The approximation error is lower than 10^-14 ^(data not shown). Therefore the integration using Gaussian quadrature is a good alternate method of the dynamic programming of hit integration, whereas the integration using Riemann sum is inadequate to be used in practical because it needs much more computing time.

### Identification of optimal seeds

In this subsection, we identify optimal spaced seeds under hit integration. Then the performance of this model is compared with those of the other probability models which compute sensitivities in their own way. On a random region with length 64, 46,252 seeds of weight 11 and length at most 20 are studied by three probability models to measure sensitivities: hit integration model, PatternHunter's model, and markov model for non-coding regions [5]. One thing to note is that both hit integration and PatternHunter are based on Bernoulli random sequence. The models based on Bernoulli random sequence has a characteristic of estimating a seed and its reversed seed with the same sensitivity. Therefore we selected the 46,252 seeds in order not to include the reversed seeds. We tested three ranges of similarity levels for hit integration: *p *= 0~1, 0.5~1, 0.3~0.7. The first range covers all similarity levels, from 0 to 1. It is selected for getting the performance of *similarity-level-free *sensitivity. The second is selected to evaluate the hit integration over the actual similarity range of the genome alignments, from 0.5 to 1, in accordance with the biological data used in the experiment. The last range, 0.3 to 0.7, is the range which shows the best average performance among several range of similarity levels. These three instances are indicated as *HI *[0, 1], *HI *[0.5, 1], and *HI *[0.3, 0.7] respectively. PatternHunter's model is equal to the hit probability at a similarity level 0.7. It can be denoted as *PH *[0.7] which means PatternHunter's model at similarity level 0.7. To indicate briefly the last model, markov model for non-coding regions will be denoted as *Markov*.

#### Sensitivities of the identified optimal seeds

We identified the optimal seeds by computing sensitivities according to hit integration and compared the sensitivities calculated by different probability models. Table 1 shows top 5 optimal seeds according to a hit integration *HI *[0, 1] and four additional seeds which are chosen from the other models. The sensitivities of the seeds listed in the table 1 are computed by the five evaluation measures: *HI *[0, 1], *HI *[0.5, 1], *HI *[0.3, 0.7], *PH *[0.7], and *Markov*. The three measures of hit integration *HI *[0, 1], *HI *[0.5, 1], *HI *[0.3, 0.7] were used to evaluate the sensitivities with the dynamic programming of hit integration. The measure of PatternHunter *PH *[0.7] was used to estimate the sensitivities with the dynamic programming of hit probability. As the measure of *Markov*, we used the latest version(1.1.1) of *mandala *provided by Buhler et al. [5]. It is a sensitivity estimation software based on markov model, and we used 5th-order non-coding model which is included in the software.

**Table 1:** Top 5 optimal spaced seeds.

seed	*HI *[0,1]	*HI *[0.5, 1]	*HI *[0.3, 0.7]	*PH *[0.7]	Markov
111**1*11**1*1*111^*a*^	0.300273 (1)	0.598730 (1)	0.0875373 (6)	0.466982 (2)	0.68499 (2482)
111*1**1*1**11*111^*b*^	0.300265 (2)	0.598713 (2)	0.0876001 (3)	0.467122 (1)	0.68869 (1425)
11*1*1*11**1**1111	0.300064 (3)	0.598314 (3)	0.0874004 (12)	0.466131 (3)	0.68301 (3359)
111**11*1**1*1*111	0.300031 (4)	0.598247 (4)	0.0873905 (13)	0.466015 (4)	0.68588 (2145)
111*1**1*111*111^*c*^	0.300031 (5)	0.598204 (6)	0.0876591 (1)	0.465521 (13)	0.70225 (52)
1111*111*1111^*d*^	0.2950 (22472)	0.5882 (22829)	0.0832 (19513)	0.4421 (25033)	0.7094 (1)
1111*111**1*111^*e*^	0.2988 (470)	0.5957 (508)	0.0866 (249)	0.4596 (747)	0.7089 (3)
111**1*1**11**1*111^*f*^	0.2999 (17)	0.5980 (14)	0.0870 (65)	0.4656 (12)	0.6802 (4907)
11111111111^*g*^	0.2590 (46252)	0.5167 (46252)	0.0538 (46252)	0.3002 (46252)	0.6066 (46133)

Top 5 optimal spaced seeds with weight 11 which are identified by hit integration are listed. The 5 seeds are ordered by *HI *[0, 1]. Each value indicates the sensitivity of the seed at the same row which is determined by the sensitivity measure at the same column. The number in the parentheses indicates the rank of the seed for each measure. The descriptions of the labeled seeds are as below; *a*: the optimal seed of *HI *[0, 1] and *HI *[0.5, 1], *b*: the default seed of PatternHunter, *c*: the optimal seed of *HI *[0.3, 0.7], *d*: the optimal seed computed by *mandala*, *e*: the seed representing 5th-order non-coding markov model in [5], *f*: the seed representing 0th-order non-coding markov model in [5], *g*: the default seed of BLAST.

Noteworthy seeds of the table 1 are listed below. The first seed marked with '*a*' is the optimal seed of the hit integration *HI *[0, 1] and *HI *[0.5, 1]. The second seed marked with '*b*' is the optimal seed of PatternHunter *PH *[0.7] as well as the default seed of PatternHunter. The fifth seed marked with '*c*' is the optimal seed of the hit integration *HI *[0.3, 0.7]. Although it is ranked fifth among the top 5 seeds, it is ranked at the relatively high position in *Markov*. We added four seeds '*d ~g*' to compare with the top 5 seeds. The seed marked with '*d*' is the best seed of the 5th-order non-coding markov model using *mandala*. Seed '*e*' and '*f*' are the representative seeds in Buhler et al. [5]; the former is the seed representing 5th-order non-coding markov model in [5] and the latter is the seed representing 0th-order non-coding markov model in [5]. The last seed marked with '*g*' is the BLAST's default seed. We need to point out the difference of the seed '*d*' and '*e*' which are representing the same model. The former is computed from the software provided by Buhler et al. [5] whereas the latter is proposed by the authors' paper. We conjecture that the markov model in the software was updated after published the paper. Another notable point is the difference of the seed '*a*' and '*f*'. They have very similar forms such that the latter has one additional don't care position at 13 by comparison with the former. However the performance of the two seeds are not similar. The seed marked with '*a*' showed better performance in theory and in practice than the seed '*f*'.

We compared the ranking of the seeds for each model because the sensitivities between models are so different to compare quantitatively. The seeds which are ranked high in hit integration are also ranked high in PatternHunter's model. It is caused by the fact that both models compute sensitivity based on Bernoulli random sequence. Although *HI *[0, 1] and *PH *[0.7] gave similar ranks to the seeds, they chose different seeds as an optimal seed. Despite of the wide superior range of similarity levels, *PH *[0.7]'s optimal seed was ranked second under *HI *[0, 1]. Therefore, considering all range of similarity levels, it reflects the cumulative sensitivity of *PH *[0.7]'s optimal seed is lower than that of *HI *[0, 1]'s optimal seed. *Markov *model showed the very different estimation ranks comparing to the models based on Bernoulli random sequence. The *Markov *model usually adapts to some domain-specific information. Thus, its performance is dependant on the contents of the biological data. It means that the performance is fluctuated by different data sets. We ascertained the existence of the fluctuation from the experimental results.

#### Testing on biological data

**Figure 2:**
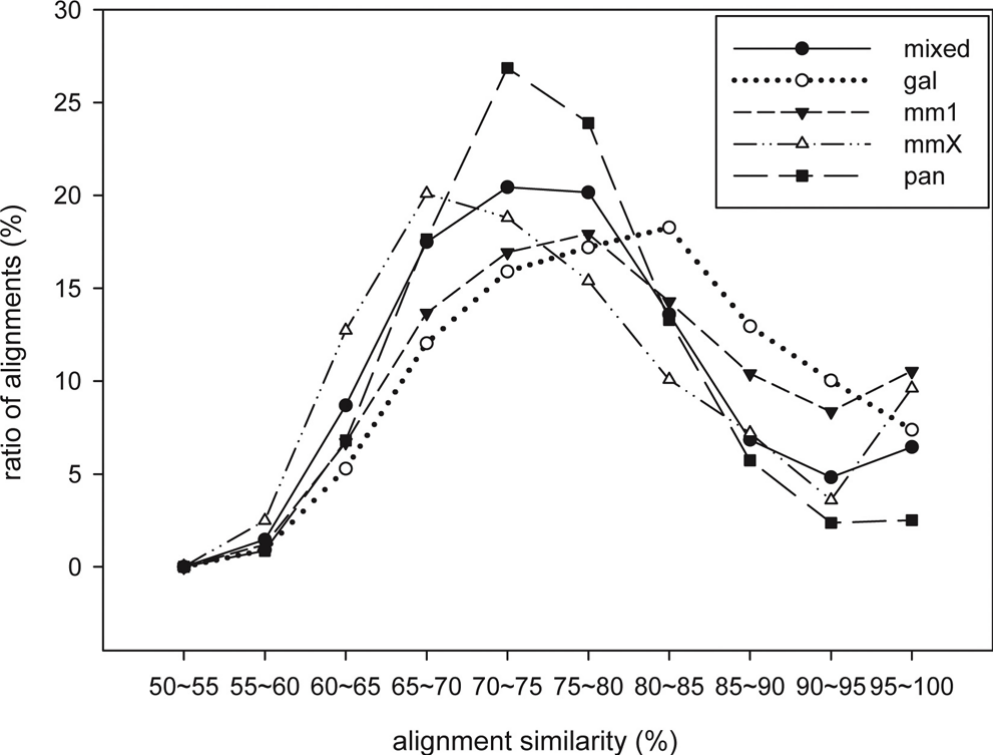
**Distributions of biological data alignments**. The distributions of the similarities of five biological data alignments are plotted by scale of 5%: *mmX*, *mm1*, *gal*, *pan*, and *mixed *(see Method).

To validate that the optimal seeds show really good performance for real biological data, we computed for each seed how many alignments from the biological data set contain at least a hit of the seed. It means that the alignment can be potentially found by an alignment program using this seed. Therefore the percent of the hit occurrences for a seed from a set of biological data is considered as the experimental sensitivity for the seed. We tested the optimal seeds of the evaluation models on the following five biological data sets: *mm*1 and *mmX *are selected from human and mouse genomes, *gal *is selected from human and chicken genomes, *pan *is selected from human and chimp genomes, and *mixed *is the cumulative data set of the four prior data sets. As we want to test the performance of the optimal seeds for similarity search in various genomic comparison, we randomly chose the data sets at the alignments of the genomes which have various genetic distances (from human ~ chimp to human ~ chicken). The similarities of the alignments are distributed widely over the similarity levels from 50% to 100% (see Figure 2). Detailed explanation of the data sets and the experimental process is described in Method.

Table 2 presents the experimental results of the seeds labeled in the table 1. Considering all data sets, the average performance of the seeds exhibits that the optimal seeds under hit integration model are better than the other models' seeds. The seed '*a*' which is the optimal under *HI *[0, 1] and *HI *[0.5, 1] shows the highest sensitivity among the five seeds. The seed '*c*' which is the optimal under *HI *[0.3, 0.7] is ranked the second. The optimal seed under PatternHunter's model marked with '*b*' shows the fifth performance among the seeds. The optimal seed under *Markov *model marked with '*d*' is ranked at the third. Another seed based on *Markov *model marked with '*e*' is ranked at the fourth. Because both '*d*' and '*e*' are selected from the 5th-order non-coding markov model, we conjecture that 5th-order markov model may be better than PatternHunter in practice.

**Table 2:** Experimental sensitivities of the optimal seeds.

seed	mixed	gal	mm1	mmX	pan	average
111**1*11**1*1*111^*a*^	0.73837 (1)	0.78102 (2)	0.79375 (2)	0.71692 (1)	0.71909 (3)	0.74983 (1)
111*1**1*1**11*111^*b*^	0.71615 (5)	0.77729 (3)	0.76228 (6)	0.69906 (2)	0.69820 (6)	0.73060 (5)
111*1**1*111*111^*c*^	0.73103 (2)	0.77070 (4)	0.80484 (1)	0.69386 (3)	0.71802 (4)	0.74369 (2)
1111*111*1111^*d*^	0.72246 (4)	0.78234 (1)	0.78821 (3)	0.67856 (5)	0.72535 (1)	0.73939 (3)
1111*111**1*111^*e*^	0.72300 (3)	0.76938 (6)	0.78317 (4)	0.68591 (4)	0.72156 (2)	0.73660 (4)
111**1*1**11**1*111^*f*^	0.71110 (6)	0.76719 (5)	0.77384 (5)	0.67652 (6)	0.70356 (5)	0.72644 (6)
11111111111^*g*^	0.60068 (7)	0.70459 (7)	0.66819 (7)	0.57455 (7)	0.57556 (7)	0.62471 (7)

The experimental sensitivities of the optimal seeds are calculated from the five biological data sets(*mixed*, *gal*, *mm*1, *mmX*, and *pan*). The tested seeds are listed as below: *a*: the optimal seed of *HI *[0, 1] and *HI *[0.5, 1], *b*: the default seed of PatternHunter, *c*: the optimal seed of *HI *[0.3, 0.7], *d*: the optimal seed computed by *mandala*, *e*: the seed representing 5th-order non-coding markov model in [5], *f*: the seed representing 0th-order non-coding markov model in [5], *g*: the default seed of BLAST.

The seed '*a*' is the optimal seed in considering all data sets because it is ranked first or second for all data sets. The '*a*'s average sensitivity is superior to that of the PatternHunter's seed '*b*' by the amount of 2% and that of the BLAST's seed '*e*' up to 12.5%. Another seed of hit integration marked with '*c*' shows also good performance against the seeds of the other models. In spite of the regular superiority of hit integration, *Markov *model's seeds '*d*' and '*e*' show irregular performance according to data sets. The seed '*d*' records the best average sensitivities for *gal *and *pan *data sets, but shows the worst sensitivities for *mmX *data sets except the consecutive seed. It is even worse than the seed '*b*' for this data sets. Consequently, the seed chosen by hit integration shows good performance for all data sets, and outperforms both the seed chosen by hit probability and the BLAST's seed. In a word, hit integration shows regularly good performance for real data sets compared with PatternHunter and *Markov *model.

### Quantitative comparison of seed sensitivity

A recent work by Mak and Benson [16] presented the notion of dominance between a class of seeds with the same weight and length. This work showed that the similarity levels can be partitioned by two or more dominant seeds. For instance, a seed is said to *dominate *another seed if the former hits at least as many homologous regions as the latter. In this situation, the latter can never be an optimal seed. Mak and Benson also proposed a method to determine the dominant ranges of similarity levels by comparing a seed with another. However, they did not show how much dominant the seed is. Hit integration enables quantitative evaluation of the seeds over the range of similarity levels.

Table 1 in Additional file 2 lists the five optimal seeds proposed by Mak and Benson [16] and the range of similiarity levels where they are optimal for the seeds with length 18 and weight 11. Seed *A *(111*1**11*1*1**111) is dominant at the similarity levels of [0, 0.05], seed *B *(111*1*1**11*1**111) is dominant at the similarity levels of [0.05, 0.07] and [0.73, 0.98], seed *C *(111*11**1*1**1*111) is dominant at [0.07, 0.73], seed *D *(11**111*1**1*111*1) is dominant at [0.98, 0.999], and seed *E *(1111*1*11**1***111) is dominant at [0.999, 1.0]. A notable point is that two seeds *B *and *C *belong to the optimal seeds presented in Table 1. Seed *B *is the optimal seed under hit integration represented the seed marked with '*a*' and seed *C *is the default seed of PatternHunter which is the seed marked with '*b*'. According to the table 1 in Additional file 1, which shows the hit integrations of the five seeds, seed *C *is the best seed by *HI *[0.07, 0.73] while seed *B *is best by *HI *[0.73, 0.98]. This complies with the work of Mak and Benson. At the similarity levels of [0.07, 0.73], seed *C *is better than seed *B *by the amount of 0.00004 and than seed *D *by 0.00067 respectively. In the same way, seed *B *is better than seed *C *by the amount of 0.00014 and than seed *D *by 0.00135 at [0.73, 0.98].

**Figure 3:**
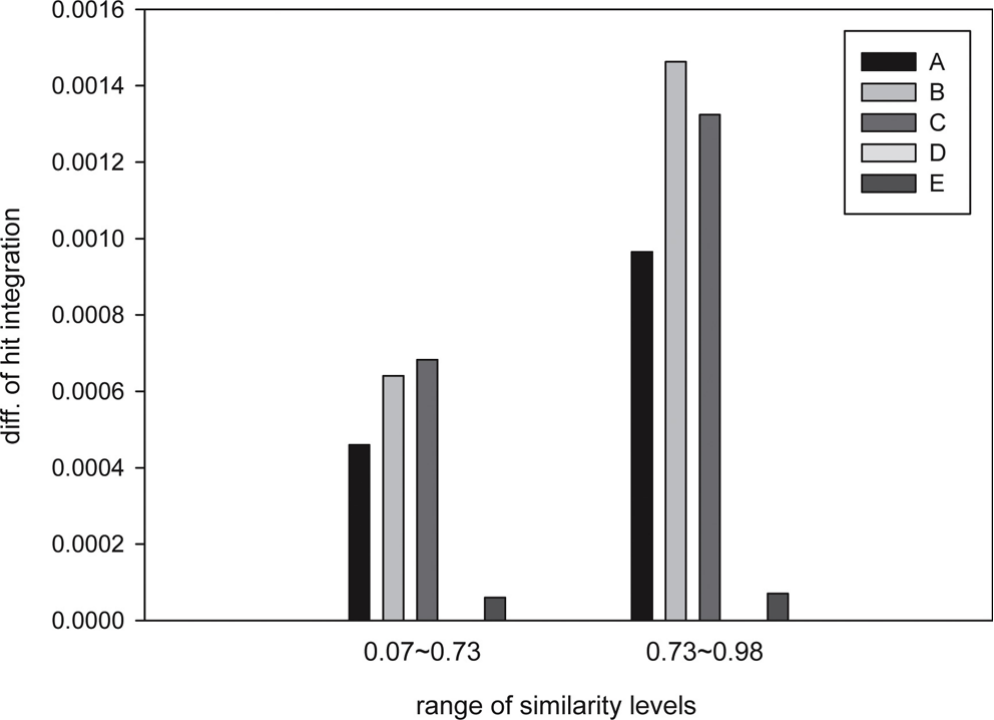
**The comparison of quantitative differences**. The quantitative differences of hit integrations for five dominant seeds are compared each other: *A*: 111*1**11*1*1**111, *B*: 111*1*1**11*1**111, *C*: 111*11**1*1**1*111, *D*: 11**111*1**1*111*1, and *E*: 1111*1*11**1***111. The value of seed *D *is set to be 0 because it showed the lowest probability at all ranges. The values of the others are subtracted by the value of the seed *D*.

When hit integration is presented as a graph, it can be easily known which is the best seed at a specific range. Figure 3 shows the quantitative comparison of the five seeds at two dominant ranges, 0.07~0.73 and 0.73~0.98. Because the differences of the other ranges are too small to compare graphically, we omitted them in the figure. In order to see the results more clearly, the lowest probability at each range is set to be 0 and the others are substituted by the lowest probability. A noteworthy observation is that the amount of the difference between seed *B *an *C *at the range of 0.07~0.73 is much smaller than at the range of 0.73~0.98. although the dominant range of seed *C *is much larger than that of seed *B*. Therefore, it can be concluded that the optimal seed under hit integration is a better seed in practical use than the PatternHunter's seed since the former's performance is much better than the latter's over the entire range of similarity levels and the range of actual biological data.

## Conclusion

We introduced hit integration, a novel idea of estimating the sensitivity of a seed at a range of similarity levels, and proposed a new algorithm of computing hit integration which is equivalent to the accumulation of hit probabilities at a range of similarity levels. Hit integration is successfully formulated as the integration of hit probability. From this formula, a dynamic programming algorithm of hit integration was developed. We identified the optimal seeds for hit integration and evaluated them for real biological data. In our experiments we studied 46,252 seeds of weight 11 and length at most 20. Through experimental studies of real biological data, the optimal seed recommended by hit integration, 111**1*11**1*1*111, showed better sensitivity than PatternHunter's seed. Moreover the average sensitivity of this seed was always superior to the PatternHunter's seed. An alternative evaluation measure, 5th-order markov model for non-coding regions, showed fluctuating performance depending on the different data set. Therefore, the optimal seed of hit integration is really optimal when the the similarity level of homology region is unknown.

Although we presented an exact algorithm of evaluation hit integration of a seed, it has a room to improve and optimize. The computing time increases quickly as the number of don't cares in the seed increases. We are now studying for more efficient algorithm to accelerate the computing time without loss of information. Hit integration is a newly proposed measure of seed's sensitivity. Therefore we expect that many possible extensions and applications for hit integration will be studied in near future.

## Methods

### Experimental process

Hit integration and the other models are evaluated on real biological data sets. For the evaluation of hit integration, five data sets for genome alignments are prepared; *mmX*: A 6.9 Mbps segment in Human chromosome X (103.6 M~110.5 M) and 6.4 Mbps segment in Mouse chromosome X (134 M~140.3 M), *mm1*: A 6 Mbps segment in Human chromosome 1 (61 M~67 M) and 5.5 Mbps segment in Mouse chromosome 4 (97 M~102 M), *gal*: A 6 Mbps segment in Human chromosome 1 (61 M~67 M) and 1.7 Mbps segment in Chicken chromosome 8 (27.7 M~29.4 M), *pan*: A 3.6 Mbps segment in Human chromosome 6 (7 M~10.6 M) and 3.5 Mbps segment in Chimp chromosome 6 (7.2 M~10.7 M), *mixed*:A 16.5 Mbps mixed segment of above four segments in Human genome and 17.1 Mbps mixed segment of above four segments in the other genomes. According to Figure 2, the similarities of the alignments are distributed normally from 50% to 100%. The peak points of the data sets are spreaded widely such as the similarity of the peak point for *mmX *is about 65% and for *gal *is about 80%. These data sets are downloaded from GenomeBrowser [29]. The downloaded versions of genome projects are: Human genome (version 18), mouse genome (version 9), chicken genome (version 3), and chimp genome (version 2).

Alignments are found using PatternHunter2 [11] with options -*phmaskj *and -*W 7*. The first option is used to filter repeats. The second option forces to find the most sensitive alignments in this program. Human genome is considered as a database and the other genome is considered as a query. Then, the resulting alignments are converted into the binary strings respectively. The matched positions are marked with 1 while the mismatched positions are marked with 0. The binary string is considered as the homologous region by its definition described in subsection 'Notation'. To evaluate the seed performance, we compute for each seed how many alignments from the data set contain a hit of the seed. We conduct an exhausted search to find a hit in a homologous region (a binary string of an alignment) by sliding a seed from the start of the region to the end. If at least a hit is occurred, we determine that this alignment can be potentially found by an alignment program using the seed. We compute the ratio of the number of the alignments which are hit by the seed against the number of all alignments. It is considered as the experimental sensitivity of the seed on a set of alignments.

## Competing interests

The authors declare that they have no competing interests.

## Authors' contributions

Chung conceived the original mathematical idea and carried out model building and empirical analysis. Park initiated, supervised and coordinated the project. All authors wrote the manuscript and approved the final version.

## Supplementary Material

Additional file 1 **Mathematical proofs for hit integration**. Mathematical proofs of Equation (7)~(10) are described in the additional file 1. Click here for file

Additional file 2 **Hit integrations of five dominant seeds**. Hit integration values for five dominant seeds are described in their ranges of similarity levels. Click here for file
