# Supplementary material for: Hit integration for identifying optimal spaced seeds
Source: BMC Bioinformatics. 2010 Jan 18;11(Suppl 1):S37. doi: 10.1186/1471-2105-11-S1-S37 (PMC3009509; doi:10.1186/1471-2105-11-S1-S37)
Supplement: Additional file 1 — Mathematical proofs for hit integration. Mathematical proofs of Equation (7)~(10) are described in the additional file 1. [file 1471-2105-11-S1-S37-S1.pdf]

Additional file 1

Mathematical Proofs for Hit Integration

We prove three lemmas used for Equation (7) $\sim$ Equation (10); The first lemma is for Equation (7), the second is for Equation (8) and Equation (9), and the third is for Equation (10).

Lemma 1 *The function I can be solved by a recursive function such as*

$$I^n[i,b] = I^n[i,0b] + I^{(n+1)}[i,1b] - I^{(n+1)}[i,0b].$$

Proof) By applying Equation (3), the right terms are derived directly.

$$\begin{aligned}
I^n[i,b] &= \int p^n f[i,b]dp \\
&= \int p^n \left(p \cdot f[i,1b] + (1-p)f[i,0b]\right) dp \\
&= \int p^{(n+1)} f[i,1b]dp + \int p^n f[1,0b]dp \\
&\quad - \int p^{(n+1)} f[i,0b]dp \\
&= I^{(n+1)}[i,1b] + I^n[i,0b] - I^{(n+1)}[i,0b].
\end{aligned}$$

Lemma 2 *If Q hits b, $I^n[i,b]$ is represented as*

$$I^n[i,b] = \begin{cases} \frac{p^{n+1}}{n+1} & if \;\; b \in B \;\; and \;\; |b| = m \\ 0 & if \;\; i < m. \end{cases}$$

Proof) Applying Equation (1) and Equation (2) respectively, the right terms are derived.

$$\begin{aligned}
I^n[i,b] &= \int p^n f[i,b]dp = \int (p^n \cdot 1)dp = \frac{p^{n+1}}{n+1} \\
I^n[i,b] &= \int p^n f[i,b]dp = \int (p^n \cdot 0)dp = 0.
\end{aligned}$$

Lemma 3 $I^n[i,0b] = I^n[i-|b|+|b'|,0b']$ *where* $0b' = B(0b)$

Proof) Applying Equation (4), the right term is derived.

$$\begin{aligned}
I^n[i,0b] &- I^n[i-|b|+|b'|,0b'] \\
&= \int p^n f[i,0b]dp - \int p^n f[i-|b|+|b'|,0b']dp \\
&= \int (p^n f[i,0b] - p^n f[i-|b|+|b'|,0b'])dp \\
&= \int p^n (f[i,0b] - f[i-|b|+|b'|,0b'])dp
\end{aligned}$$

$$
\begin{aligned}
&= \int p^n \cdot 0 \; dp \\
&= 0.
\end{aligned}
$$
