# Supplementary material for: Hit integration for identifying optimal spaced seeds
Source: BMC Bioinformatics. 2010 Jan 18;11(Suppl 1):S37. doi: 10.1186/1471-2105-11-S1-S37 (PMC3009509; doi:10.1186/1471-2105-11-S1-S37)
Supplement: Additional file 2 — Hit integrations of five dominant seeds. Hit integration values for five dominant seeds are described in their ranges of similarity levels. [file 1471-2105-11-S1-S37-S2.pdf]

Additional file 2

Hit integrations of five dominant seeds

Table 1: Hit integrations of five dominant seeds: A: 111*1**11*1*1**111, B: 111*1*1**11*1**111, C: 111*11**1*1**1*111, D: 11**111*1**1*111*1, E: 1111*1*11**1***111. '*' marked seed is the seed that shows the highest hit integration value for each similarity range.

similarity range	seed	hit integration
0 ∼ 0.0524790924	A*	3.256612342841852E-14
	B	3.256612342749398E-14
	C	3.256612341798721E-14
	D	3.256612298819122E-14
	E	3.2566115293013535E-14
0.0524790924 ∼ 0.0775105071	A	7.289693279684254E-12
	B*	7.289693295244111E-12
	C	7.289693292109644E-12
	D	7.2896922736172E-12
	E	7.289680997729342E-12
0.0775105071 ∼ 0.7304317142	A	0.0784639860717529
	B	0.07864496902275496
	C*	0.07868695000938536
	D	0.07800419490374307
	E	0.07806383405843921
0.7304317142 ∼ 0.9845899783	A	0.9182755095478847
	B*	0.9187732881727378
	C	0.9186347185335761
	D	0.9173105651704655
	E	0.9173812480420739
0.9845899783 ∼ 0.9997355115	A	0.9999999999145007
	B	0.9999999998955471
	C	0.9999999998159751
	D*	0.9999999999357159
	E	0.999999999871687
0.9997355115 ∼ 1.0	A	0.9999999986121215
	B	0.9999999985951211
	C	0.9999999990853576
	D	0.9999999979370978
	E*	0.9999999993844448
